# Publisher Correction: Multiscale architecture design of 3D printed biodegradable Zn-based porous scaffolds for immunomodulatory osteogenesis

**DOI:** 10.1038/s41467-024-48079-6

**Published:** 2024-04-29

**Authors:** Shuang Li, Hongtao Yang, Xinhua Qu, Yu Qin, Aobo Liu, Guo Bao, He Huang, Chaoyang Sun, Jiabao Dai, Junlong Tan, Jiahui Shi, Yan Guan, Wei Pan, Xuenan Gu, Bo Jia, Peng Wen, Xiaogang Wang, Yufeng Zheng

**Affiliations:** 1 School of Engineering Medicine, School of Biological Science and Medical Engineering, Beihang University, 100191 Beijing, China; 2 School of Materials Science and Engineering, Peking University, 100871 Beijing, China; 3 Department of Bone and Joint Surgery, Department of Orthopedics, Renji Hospital, Shanghai Jiao Tong University School of Medicine, 200001 Shanghai, China; 4 Department of Mechanical Engineering, Tsinghua University, 100084 Beijing, China; 5 Department of Reproduction and Physiology National Research Institute for Family Planning, 100081 Beijing, China; 6 School of Materials Science and Engineering, Zhengzhou University, 450003 Zhengzhou, China; 7 College of Chemistry and Molecular Engineering, Peking University, 100871 Beijing, China

**Keywords:** Implants, Biomedical materials

Correction to: *Nature Communications* 10.1038/s41467-024-47189-5, published online 11 April 2024

The original version of this Article contained an error in Fig. 4. The submitted and peer reviewed versions of this Article contained two distinct “MC3T3-E1” graphs in Fig. 4c, however one of those graphs was inadvertently duplicated during the production process. The correct version of Fig. 4 is:
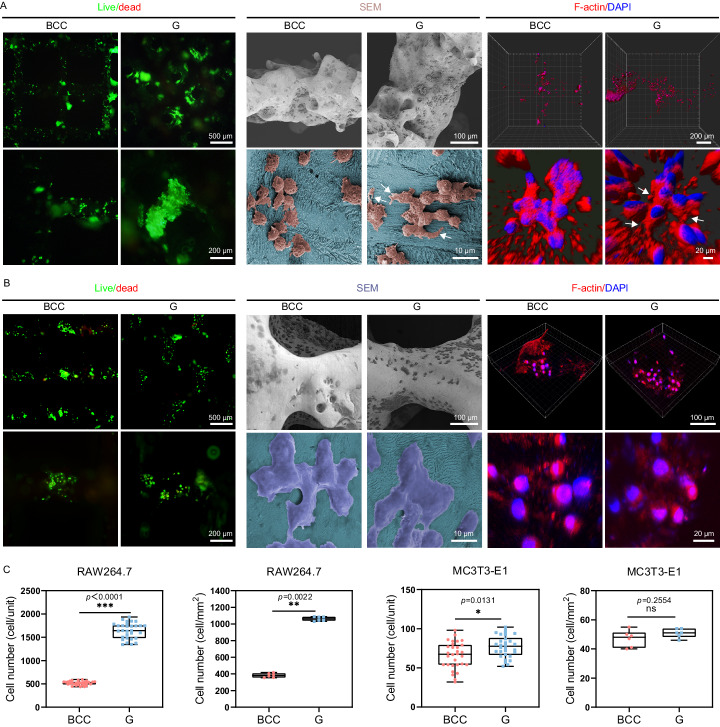


This has been corrected in both the PDF and HTML versions of the Article.

