# Supplementary material for: Multiscale architecture design of 3D printed biodegradable Zn-based porous scaffolds for immunomodulatory osteogenesis
Source: Nat Commun. 2024 Apr 11;15:3131. doi: 10.1038/s41467-024-47189-5 (PMC11009309; doi:10.1038/s41467-024-47189-5)
Supplement: Supplementary file 1 — Supplementary information [file 41467_2024_47189_MOESM1_ESM.pdf]

Supplementary Information for

Multiscale architecture design of 3D printed biodegradable Zn-based porous scaffolds for immunomodulatory osteogenesis

Authors list

Shuang Li[1†], Hongtao Yang[1,2†, *], Xinhua Qu[3†], Yu Qin[2], Aobo Liu[4], Guo Bao[5], He Huang[6], Chaoyang Sun[1], Jiabao Dai[4], Junlong Tan[1], Jiahui Shi[2], Yan Guan[7], Wei Pan[7], Xunan Gu[1], Bo Jia[4], Peng Wen[4*], Xiaogang Wang[1*], Yufeng Zheng[2*]

Affiliations

[1]School of Engineering Medicine, School of Biological Science and Medical Engineering, Beihang University, Beijing 100191, China
[2]School of Materials Science and Engineering, Peking University, Beijing, 100871, China
[3]Department of Bone and Joint Surgery, Department of Orthopedics, Renji Hospital, Shanghai Jiao Tong University School of Medicine, Shanghai 200001, China
[4]Department of Mechanical Engineering, Tsinghua University, Beijing, 100084, China.
[5]Department of Reproduction and Physiology National Research Institute for Family Planning Beijing 100081, China
[6]School of Materials Science and Engineering, Zhengzhou University, Zhengzhou, 450003, China
[7]College of Chemistry and Molecular Engineering, Peking University, Beijing, 100871, China

† These authors contributed equally to this work.
Corresponding author: Hongtao Yang, Yufeng Zheng
Email address: yang276070@buaa.edu.cn, yfzheng@pku.edu.cn

This PDF file includes:

Figures. S1 to S9
Tables S1 to S5

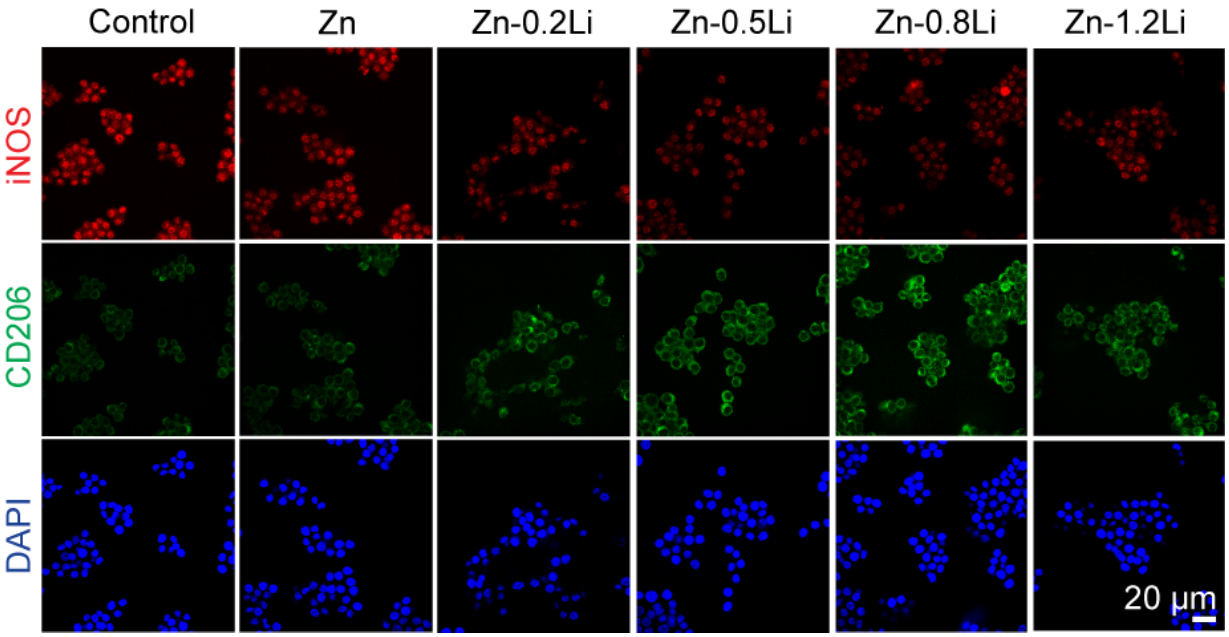

Fig. S1. Immunofluorescence staining of iNOS, CD206, and DAPI of RAW264.7 after co-culture with material extracts for 48h. Each image was acquired independently three times, with similar results.

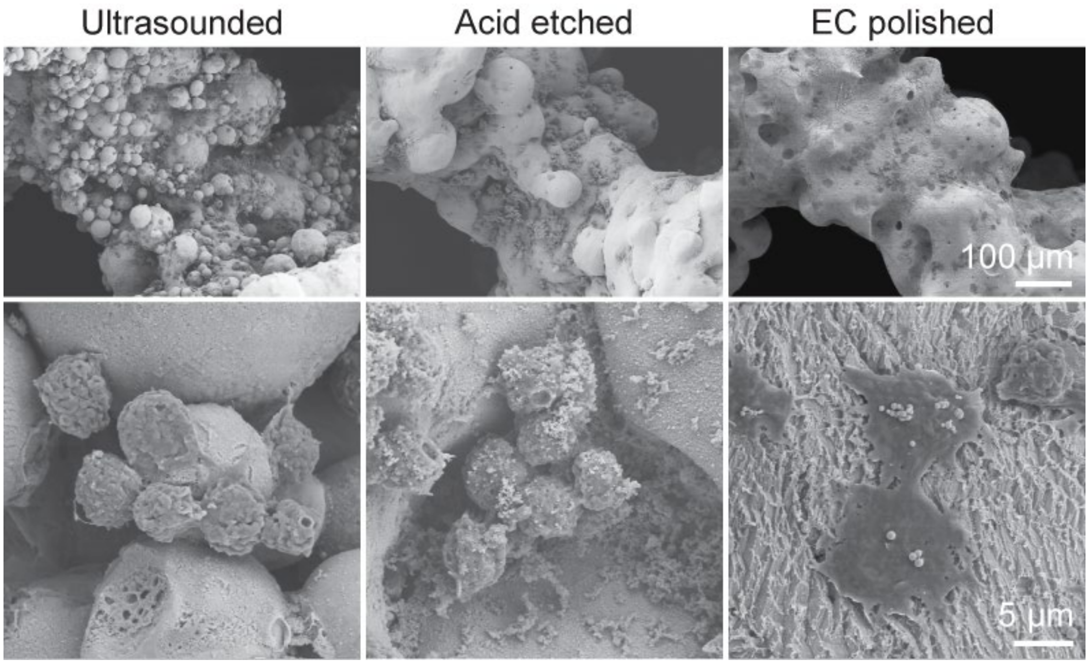

Fig. S2. SEM images of RAW264.7 cells after 6h attachment on different surfaces. Each image was acquired independently three times, with similar results.

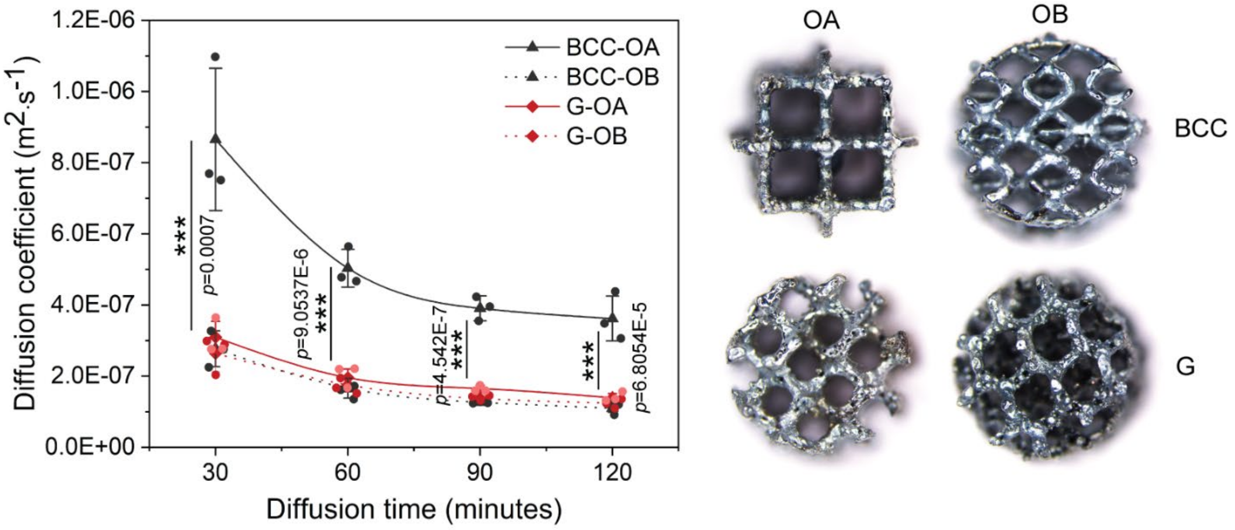

Fig. S3. Diffusion coefficient of Zn ions in scaffolds with BCC and G unit over 120 minutes (n=3, independent experiments). Data are presented as mean ± standard deviation. OA: orientation A, OB: orientation B. P-values are calculated using one-way ANOVA with Tukey's post hoc test, *p < 0.05, **p < 0.01, ***p < 0.005.

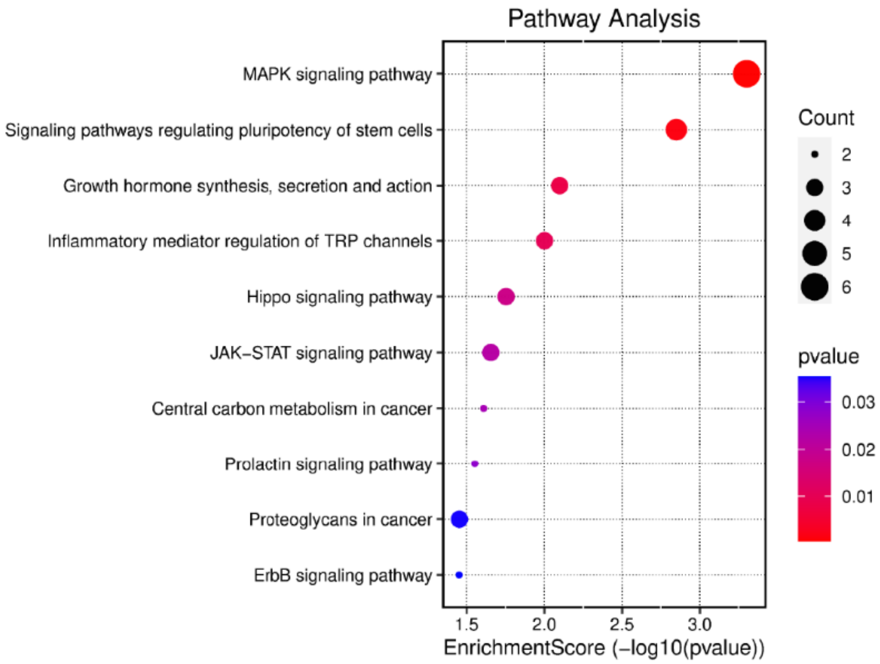

Fig. S4. Enriched KEGG pathway of G scaffold versus control.

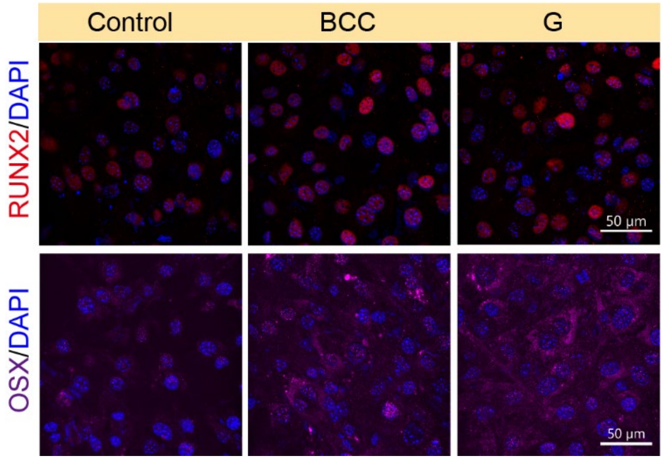

Fig. S5. Runx2 and Osx immunofluorescence staining of MC3T3-E1 cultured in conditioned medium at 7 days. Each image was acquired independently three times, with similar results.

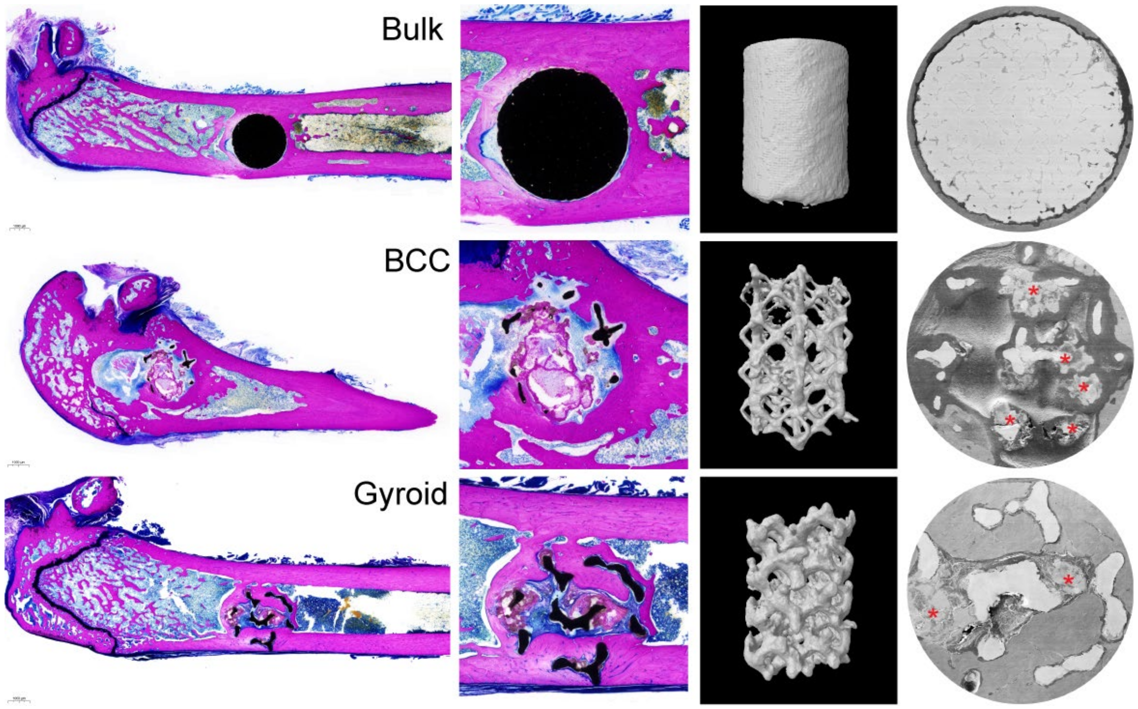

Fig. S6. Comparison of new bone regeneration and degradation between Zn-Li alloy bulk sample and scaffolds at 3 months. Red asterisks indicate scaffold degradation. Each image was acquired independently three times, with similar results.

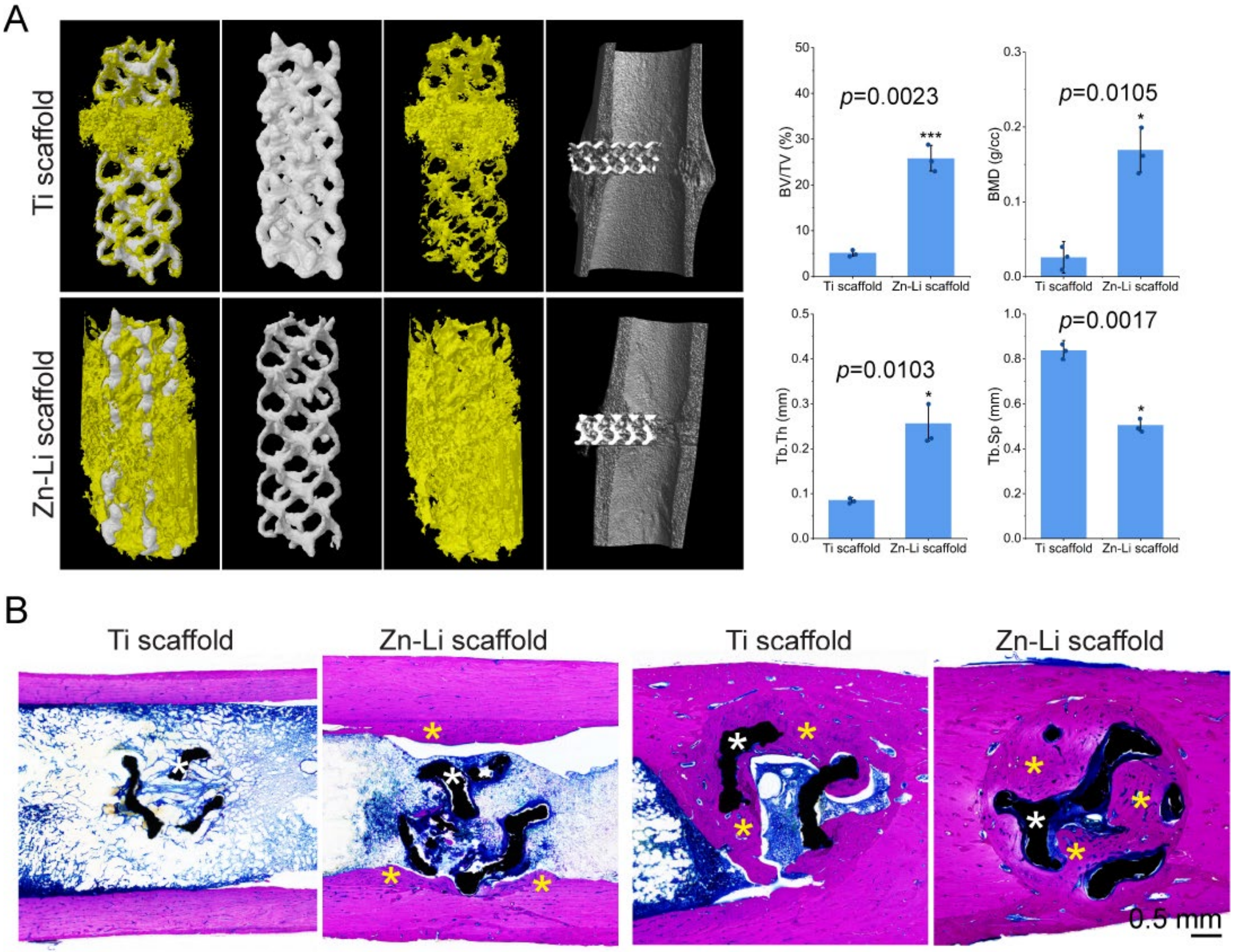

Fig. S7. Bone regeneration comparison between Zn-Li scaffold and Ti scaffold in a critical bone defect rabbit model at 2 months. A Micro-CT reconstruction of new bone tissue and metallic implants with quantitative analysis (*n*=3, independent experiments) of bone volume/tissue volume (BV/TV), bone mineral density (BMD), trabecular thickness (Tb. Th), and trabecular separation (Tb. Sp). New bone is marked in yellow, implants are marked in white. B Methylene blue acid fuchsin staining of bone defect regions. Yellow asterisks indicate newly formed bone, white asterisks are scaffold struts. Data are presented as mean ± standard deviation. P-values are calculated using one-way ANOVA with Tukey's post hoc test, $*p < 0.05$, $**p < 0.01$, $***p < 0.005$. Each image was acquired independently three times, with similar results.

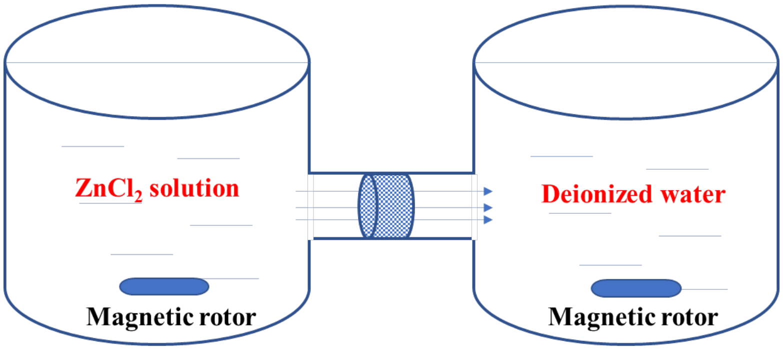

Fig. S8. Schematic diagram of diffusion device.

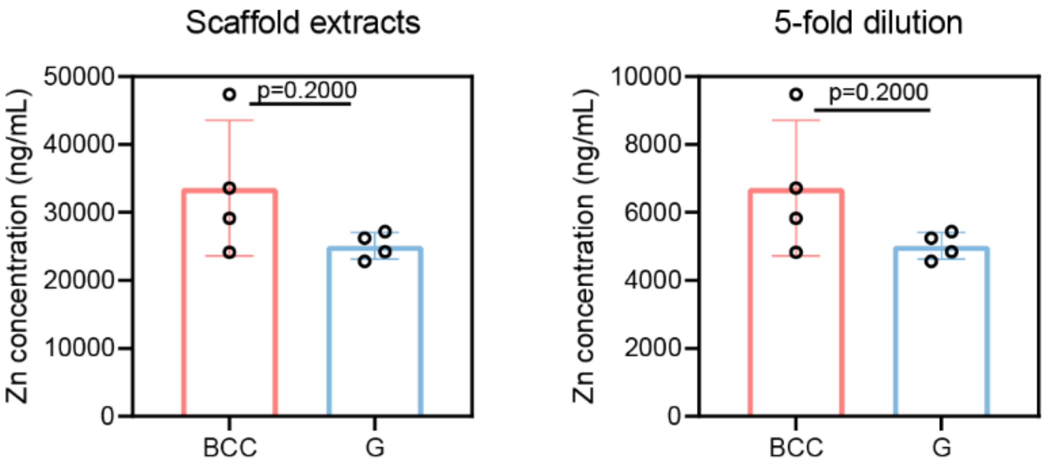

Fig. S9. Zn concentrations of BCC and G scaffold extracts determined by ICP (n=4 independent experiments). Data are presented as mean ± standard deviation. P-values are calculated using one-way ANOVA unpaired t-test with a Mann-Whitney test.

Table S1 Chemical composition of Zn-Li alloys

Nominal composition (wt.%)	Actual composition (wt.%)
Zn-0.2Li	0.239
Zn-0.5Li	0.494
Zn-0.8Li	0.779
Zn-1.2Li	1.36

Table S2. Binding energy of corrosion products detected in Zn-Li alloys after immersion in SBF

Products	Component	Binding energy (eV)	
		Measured	Ref.
ZnO	Zn 2$p_{3/2}$	1021.8	1021.9
	O 1*s*	530.6	530.8
	Li 1*s*	55.0	55.1
Li_2CO_3	C 1*s*	289.6	289.6
	O 1*s*	530.6	531.4

Table S3. CT-measured structure parameters of Zn-Li scaffolds with different pore unit and size.

Size	Structure characteristics	BCC	G
Φ3×4 mm	Porosity (%)	88	86
	Pore size (mm)	0.75	0.4
	Strut thickness (mm)	0.4	0.5
	Surface area (mm^2)	78.7	76.08
	Scaffold volume (mm^3)	3.58	3.99
	Specific surface area (scaffold) (mm^{-1})	21.99	19.04
Φ10×2 mm	Porosity (%)	90.48	90.34
	Surface area (mm^2)	404.12	353.59
	Volume (mm^3)	15.20	15.14
	Specific surface area (scaffold)	26.6	23.35

Table S4 Comparison of key properties between pure Ti, autologous bone and Zn-Li alloys

Materials	Mechanical properties				Biodegradability	Bioactivity	Printability
	UTS[b] (MPa)	UCS[c] (MPa)	Elongation %	Elastic Modulus (GPa)			
Cortical bone[1]	50-151	130-200	-	7-30	No	Yes	No
Pure Ti (Grade 1-4) [a]	240-550	-	15-24	110	No	No	Yes
Zn-Li alloys	252-780	790-1100[d2]	0-26	100	Yes	Yes	Yes

[a]ASTM-F67
[b] Ultimate tensile strength
[c] Ultimate compressive strength
[d] Zn-Li alloys have compression super plasticity, the maximum stress before 50% compressive strain was defined as ultimate compressive strength

References

1. Gerhardt, L. C., Boccaccini, A. R. Bioactive glass and glass-ceramic scaffolds for bone tissue engineering. *Materials* **3**, 3867-3910 (2010).
2. Yang, H., Jia, B., Zhang, Z., Qu, X., Li, G., Lin, W., Zhu, D., Dai, K., Zheng, Y. Alloying design of biodegradable zinc as promising bone implants for load-bearing applications. *Nat. commun.* **11**, 1-16 (2020).

Table S5 Primer sequences of migration-related genes for qRT-PCR

Primer	Sequences
iNos	GTTCTCAGCCCAACAATACAAGA<br>GTGGACGGGTCGATGTCAC
Il-1β	GCAACTGTTCCTGAACTCAACT<br>ATCTTTTGGGGTCCGTCAACT
Tnf-α	CCCTCACACTCAGATCATCTTCT<br>GCTACGACGTGGGCTACAG
Arg1	CTCCAAGCCAAAGTCCTTAGAG<br>AGGAGCTGTCATTAGGGACATC
Il-4	GGTCTCAACCCCCAGCTAGT<br>GCCGATGATCTCTCTCAAGTGAT
Il-10	GCTCTTACTGACTGGCATGAG<br>CGCAGCTCTAGGAGCATGTG
Col1a1	GCTCCTCTTAGGGGCCACT<br>CCACGTCTCACCATTGGGG
Opg	ACCCAGAAACTGGTCATCAGC<br>CTGCAATACACACACTCATCACT
Opn	ACCCAGAAACTGGTCATCAGC<br>CTGCAATACACACACTCATCAC
Alp	CCAACTCTTTTGTGCCAGAGA<br>GGCTACATTGGTGTTGAGCTTTT
Gapdh	AGGTCGGTGTGAACGGATTTG<br>TGTAGACCATGTAGTTGAGGTCA
