# Supplementary material for: Multiscale architecture design of 3D printed biodegradable Zn-based porous scaffolds for immunomodulatory osteogenesis
Source: Nat Commun. 2024 Apr 11;15:3131. doi: 10.1038/s41467-024-47189-5 (PMC11009309; doi:10.1038/s41467-024-47189-5)
Supplement: Supplementary file 2 — Reporting Summary [file 41467_2024_47189_MOESM2_ESM.pdf]

nature portfolio

Corresponding author(s): Hongtao Yang

Last updated by author(s): Mar 18, 2024

Reporting Summary

Nature Portfolio wishes to improve the reproducibility of the work that we publish. This form provides structure for consistency and transparency in reporting. For further information on Nature Portfolio policies, see our Editorial Policies and the Editorial Policy Checklist.

Statistics

For all statistical analyses, confirm that the following items are present in the figure legend, table legend, main text, or Methods section.

n/a	Confirmed	
☐	☒	The exact sample size (*n*) for each experimental group/condition, given as a discrete number and unit of measurement
☐	☒	A statement on whether measurements were taken from distinct samples or whether the same sample was measured repeatedly
☐	☒	The statistical test(s) used AND whether they are one- or two-sided<br>*Only common tests should be described solely by name; describe more complex techniques in the Methods section.*
☒	☐	A description of all covariates tested
☒	☐	A description of any assumptions or corrections, such as tests of normality and adjustment for multiple comparisons
☐	☒	A full description of the statistical parameters including central tendency (e.g. means) or other basic estimates (e.g. regression coefficient) AND variation (e.g. standard deviation) or associated estimates of uncertainty (e.g. confidence intervals)
☐	☒	For null hypothesis testing, the test statistic (e.g. *F*, *t*, *r*) with confidence intervals, effect sizes, degrees of freedom and *P* value noted<br>*Give P values as exact values whenever suitable.*
☒	☐	For Bayesian analysis, information on the choice of priors and Markov chain Monte Carlo settings
☒	☐	For hierarchical and complex designs, identification of the appropriate level for tests and full reporting of outcomes
☒	☐	Estimates of effect sizes (e.g. Cohen's *d*, Pearson's *r*), indicating how they were calculated

Our web collection on statistics for biologists contains articles on many of the points above.

Software and code

Policy information about availability of computer code

Data collection	No software was used
Data analysis	GraphPad Prism Version 8.4 software (GraphPad Software, La Jolla CA, USA) and Origin 2019b (Originlab, Northampton MA, USA)

For manuscripts utilizing custom algorithms or software that are central to the research but not yet described in published literature, software must be made available to editors and reviewers. We strongly encourage code deposition in a community repository (e.g. GitHub). See the Nature Portfolio guidelines for submitting code & software for further information.

Data

Policy information about availability of data

All manuscripts must include a data availability statement. This statement should provide the following information, where applicable:

- Accession codes, unique identifiers, or web links for publicly available datasets
- A description of any restrictions on data availability
- For clinical datasets or third party data, please ensure that the statement adheres to our policy

The authors declare that all data supporting the findings of this study are available within this paper and its Supplementary Files. The RNA-seq data generated in this study have been deposited in the NCBI Gene Expression Ominubus (GEO) database under accession number GSE262010. Source data are provided with this paper.

Research involving human participants, their data, or biological material

Policy information about studies with human participants or human data. See also policy information about sex, gender (identity/presentation), and sexual orientation and race, ethnicity and racism.

Reporting on sex and gender	We don't have related issues
Reporting on race, ethnicity, or other socially relevant groupings	We don't have related issues
Population characteristics	We don't have related issues
Recruitment	We don't have related issues
Ethics oversight	We don't have related issues

Note that full information on the approval of the study protocol must also be provided in the manuscript.

Field-specific reporting

Please select the one below that is the best fit for your research. If you are not sure, read the appropriate sections before making your selection.

☒ Life sciences ☐ Behavioural & social sciences ☐ Ecological, evolutionary & environmental sciences

For a reference copy of the document with all sections, see nature.com/documents/nr-reporting-summary-flat.pdf

Life sciences study design

All studies must disclose on these points even when the disclosure is negative.

Sample size	The Zn-Li alloys for electrochemical test were hot extruded, and cut into 10 mm × 2 mm plates. (10 mm is one of the standardized diameters for as extruded metal rods, the commonly used thickness is 1-2 mm)<br>The samples for tensile test were prepared according to ASTM-E08.<br>The mean powder size for 3D printing is 29.7 µm. (this is the optimal powder size for Zn-Li alloy that can be fabricated to date)<br>The biomimic triple periodic minimal surface Gyroid (G) structure and traditional body-centered cubic (BCC) scaffolds for in vitro cell test and electrochemical test were printed to 10 mm × 2 mm (diameter × height). (For cell test, 10 mm diameter is suitable to fix in 24-well cell plates. The unit size for G and BCC is 1 mm, 2 mm thickness allow 2 repeating units for cell seeding)<br>The scaffolds for compression test and dynamic immersion test were printed to 6 mm × 6 mm (diameter × height) according to ASTM E9-09 and ASTM G31, respectively.<br>The scaffolds for animal implantation were printed to 3 mm × 4 mm (diameter × height). (This is the maximum size of implant for rat femoral condyle)<br>The MMA embedded slices for SEM samples were wire-cut into 2 cm × 2 cm sample (1 mm thickness). (This is the optimal size allowed for SEM imaging considering conductivity and integrity of the sample)<br>The Zn-Li alloys samples for extract liquid were cut to 10 mm × 2 mm (diameter × height). The extract liquid of the scaffolds was prepared according to ISO 10993-12.
Data exclusions	Data were not excluded from analysis.
Replication	All the quantitative data in this study has been repeated for as least 3 times independently. The n numbers are presented in each figure legend. All attempts at replication were shown with scatter in related figures.
Randomization	The samples in this experiment were randomly assigned, and there was no considered control.
Blinding	The data used for statistics in the article were measured in the same environment and there is no blinding issue.

Reporting for specific materials, systems and methods

We require information from authors about some types of materials, experimental systems and methods used in many studies. Here, indicate whether each material, system or method listed is relevant to your study. If you are not sure if a list item applies to your research, read the appropriate section before selecting a response.

Materials & experimental systems

n/a	Involved in the study	
☐	☒	Antibodies
☐	☒	Eukaryotic cell lines
☒	☐	Palaeontology and archaeology
☐	☒	Animals and other organisms
☒	☐	Clinical data
☒	☐	Dual use research of concern
☒	☐	Plants

Methods

n/a	Involved in the study	
☒	☐	ChIP-seq
☒	☐	Flow cytometry
☒	☐	MRI-based neuroimaging

Antibodies

Antibodies used	iNOS Antibody; Proteintech; 22226-1-AP; Polyclonal; IHC; IF<br>CD206 Antibody; Santa Cruz; sc-58986;Monoclonal; IF<br>CD163 Antibody; Proteintech; 16646-1-AP; Polyclonal; IHC<br>OCN Antibody; Proteintech; 23418-1-AP; Polyclonal; IHC<br>Runx2 Antibody; Cell signaling technology; #12556; Monoclonal; IF<br>Osx Antibody; Abcam; ab209484; Monoclonal; IF
Validation	iNOS Antibody:<br>https://www.ptgcn.com/products/NOS2-Antibody-22226-1-AP.htm<br>CD206 Antibody:<br>https://www.scbt.com/p/cd206-antibody-15-2?requestFrom=search<br>CD163 Antibody:<br>https://www.ptgcn.com/products/CD163-Antibody-16646-1-AP.htm<br>OCN Antibody:<br>https://www.ptgcn.com/products/Osteocalcin-Antibody-23418-1-AP.htm<br>Runx2 Antibody:<br>https://www.cellsignal.cn/products/primary-antibodies/runx2-d1l7f-rabbit-mab/12556<br>Osx Antibody:<br>https://www.abcam.cn/products/primary-antibodies/sp7--osterix-antibody-epr21034-ab209484.html

Eukaryotic cell lines

Policy information about cell lines and Sex and Gender in Research

Cell line source(s)	MC3T3-E1 and RAW264.7 cells were purchased from Wuhan Prucell Life Sciences Co. (Wuhan, China).
Authentication	Commercially purchased cell lines were not authenticated by study participants.
Mycoplasma contamination	All cell lines were tested negative for mycoplasma contamination.
Commonly misidentified lines<br>(See ICLAC register)	No commonly misidentified cell lines were used in the study.

Animals and other research organisms

Policy information about studies involving animals; ARRIVE guidelines recommended for reporting animal research, and Sex and Gender in Research

Laboratory animals	12-week-old male Sprague-Dawley rats weighing 350-400 g were used for in vivo femoral condyle implantation.
Wild animals	No wild animals were used in the study.
Reporting on sex	The male Sprague-Dawley (SD) rats were used for in vivo femoral condyle implantation.<br>There are several reasons why male SD rats are selected as subjects in scientific experiments:<br>1. Physiological differences: Female mammals have oestrous cycles that eventually turn into menstrual periods if they are not pregnant, potentially adding a tricky variable to scientific trials and data analysis. However, a considerable number of studies have also found that female mice do not respond differently to drugs at different times in the physiological cycle.<br>2. Ease of control: Male rats can be more easily controlled and standardized during the experiment because they do not have physiological changes such as the menstrual cycle.<br>3. Avoid confusion: The use of male animals can avoid the influence of physiological state changes such as estrus or pregnancy on the experimental results.
Field-collected samples	No field collected samples were used in the study.

Ethics oversight	All surgical procedures were conducted by the ARRIVE guidelines and received approval from the Animal Ethics Committee of the National Research Institute for Family Planning (NRIFH 21-2203-18).

Note that full information on the approval of the study protocol must also be provided in the manuscript.
